# A novel and efficient murine model of Bietti crystalline dystrophy

**DOI:** 10.1242/dmm.049222

**Published:** 2022-03-01

**Authors:** Yafang Wang, Yang Liu, Shu Liu, Xiaomeng Li, Xinxin Liu, Ming Jiao, Yuqin Yang, Xueting Luo, Fenghua Wang, Xiaoling Wan, Xiaodong Sun

**Affiliations:** 1 Department of Ophthalmology, Shanghai General Hospital (Shanghai First People's Hospital), Shanghai Jiao Tong University School of Medicine, 100 Haining Road, Shanghai, China 200080; 2 Shanghai Key Laboratory of Ocular Fundus Diseases, 100 Haining Road, Shanghai, China 200080; 3 Laboratory Animal Center, Shanghai General Hospital (Shanghai First People's Hospital), Shanghai Jiao Tong University School of Medicine, 650 Xinsongjiang Road, Shanghai, China 201620; 4 Shanghai Engineering Center for Visual Science and Photomedicine, 100 Haining Road, Shanghai, China 200080; 5 National Clinical Research Center for Eye Diseases, 100 Haining Road, Shanghai, China 200080; 6 Shanghai Engineering Center for Precise Diagnosis and Treatment of Eye Diseases, 100 Haining Road, Shanghai, China 200080

**Keywords:** Bietti crystalline dystrophy, *Cyp4v3*, Mouse model, Lipid accumulation, Retinal degeneration

## Abstract

Bietti crystalline dystrophy (BCD) is an autosomal recessive inherited retinal disease, resulting in blindness in most patients. The etiology and development mechanism of it remain unclear. Given the defects in previous mouse models of BCD, we generated a new *Cyp4v3^−/−^* mouse model, using CRISPR/Cas9 technology, for investigating the pathogenesis of BCD. We estimated the ocular phenotypes by fundus imaging, optical coherence tomography (OCT) and full-field scotopic electroretinography, and investigated the histological features by Hematoxylin and Eosin staining, Oil Red O staining and immunofluorescence. This model effectively exhibited age-related progression that mimicked the human ocular phenotypes. Moreover, gas chromatography-mass spectrometry and RNA-seq analysis indicated that the defect of *Cyp4v3* led to the abnormal lipid metabolism, inflammation activation and oxidative stress of retina. Notably, inflammation activation and oxidative stress could also promote the progression of BCD in light-induced retinal degeneration. In conclusion, our data provided evidence that we established a novel and more effective *Cyp4v3* knockout preclinical mouse model for BCD, which served as a useful tool for evaluating the effect of drugs and gene therapy *in vivo*.

## Abstract (precis)

**Summary:** A novel and more effective *Cyp4v3* knockout preclinical mouse model for Bietti crystalline dystrophy, which will serve as a useful tool for investigating the pathogensis of the disease *in vivo*.

## INTRODUCTION

Bietti crystalline dystrophy (BCD) is an autosomal recessive inherited retinal dystrophy that leads to irreversible vision loss, and was first reported by Gian Battista Bietti in 1937 (Bietti, 1937). It accounts for 10% of autosomal recessive retinitis pigmentosa patients and is relatively more commonly observed in Chinese and Japanese populations (Yin et al., 2016; Mataftsi et al., 2004). The disease is characterized by intraretinal and corneal yellow-white crystalline deposits, atrophy of the retinal pigment epithelium (RPE) and sclerosis of the choroidal vessels (Manzouri et al., 2012). Clinically, between the second to fourth decade of life, patients develop decreased vision, nyctalopia and paracentral scotoma (Li et al., 2004). They encounter peripheral visual field loss and develop irreversible visual impairment by the fifth or sixth decade of life (Li et al., 2004). To date, no clinical treatment for this severe disease has been developed.

BCD is caused by mutations in the cytochrome P450 family 4 subfamily V polypeptide 2 (*CYP4V2*) gene (Manzouri et al., 2012). CYP4V2 protein is widely distributed and present in the eye, and is highly expressed in the RPE cells and weakly expressed in the cornea (Nakano et al., 2009, 2012). In addition, *CYP4V2* encodes a polyunsaturated omega-3 fatty acids hydroxylase, which mediates fatty acid precursors into n-3 polyunsaturated fatty acids (n-3 PUFAs) (Nakano et al., 2009, 2012). Lymphocytes and fibroblasts of patients with BCD have abnormally high levels of triglycerides and stored cholesterol (Lee et al., 2001). Given these findings, it can be reasonable to speculate that the ocular onset presentation of BCD is related to imbalance of lipid metabolism. However, the specific pathogenesis of BCD remains unknown.

The *Cyp4v3* gene, the mouse ortholog, is highly homologous to human *CYP4V2*. Two different murine knockout models have been generated successively (Lockhart et al., 2014; Qu et al., 2020). Although both models could partially mimic the development of crystalline deposits, a hallmark of BCD, the former took as long as 6 months (Lockhart et al., 2014), and the latter needed to be administered a high-fat diet from postnatal week 4 (Qu et al., 2020). The lack of an effective BCD murine model has limited our understanding of the pathological mechanisms of BCD and the consequent evaluation of therapeutic effects *in vivo*. Here, we developed a novel and effective *Cyp4v3*^−/−^ mouse model for BCD. Through a long-term follow-up study on the natural course of the characteristics of eyes, we confirmed that this model could recapitulate the phenotype of crystalline deposits as early as postnatal week 6, followed by changes in electroretinogram (ERG) and progressive retinal degeneration. Moreover, we analyzed lipid and transcriptome profiles by gas chromatography-mass spectrometry (GC-MS) and RNA-seq, respectively. In addition, the loss of *Cyp4v3* induced microglia- and Müller cells-mediated inflammation and oxidative stress, which could also accelerate the progression of BCD in a light-induced retinal degeneration (LIRD) mouse model.

## RESULTS

### Generation of a novel *Cyp4v3* knockout mouse

**Fig. 1.**
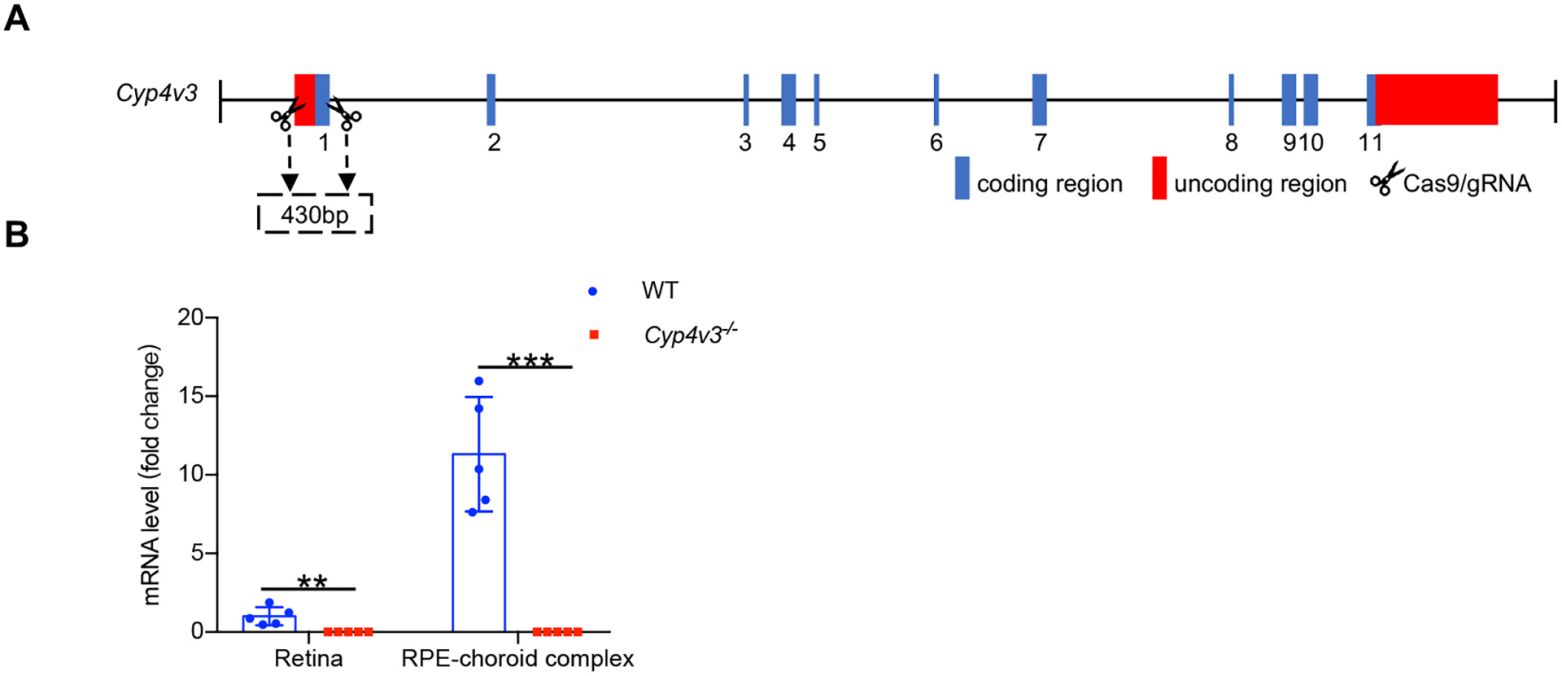
**Generation of *Cyp4v3^−/−^* mice.** (A) The gene structure of mouse *Cyp4v3* and the design strategy. NGG-terminated targets were designed near exon 1 and cleaved with CRISPR/Cas9. (B) mRNA analysis of Cyp4v3 in the retina and RPE-choroid complex. Data are mean±s.d. **P<0.01, ***P<0.001 (Student’s unpaired two-tailed *t*-test). *n*=5 for each group. WT, wild type.

Instead of deleting the entire locus of mouse *Cyp4v3* as reported previously (Lockhart et al., 2014; Qu et al., 2020), we generated *Cyp4v3* knockout mice using CRISPR/Cas9 technology targeting exon 1 of the *Cyp4v3* gene, which led to a complete loss of exon 1 (Fig. 1A). To confirm whether the knockout of *Cyp4v3* was successful, we determined the expression of Cyp4v3 by RT-qPCR. As shown in Fig. 1B, Cyp4v3 was highly expressed in the RPE-choroid complex of wild-type (WT) mice, and efficient deletion of *Cyp4v3* was verified in the retina and RPE-choroid complex of *Cyp4v3^−/−^* mice. Moreover, *Cyp4v3^−/−^* mice were born with the expected Mendelian ratios and were healthy and fertile. In short, the *Cyp4v3* gene was knocked out successfully in this animal model.

### Crystal depositions appeared as early as at 6 weeks of age in *Cyp4v3^−/−^* mice

**Fig. 2.**
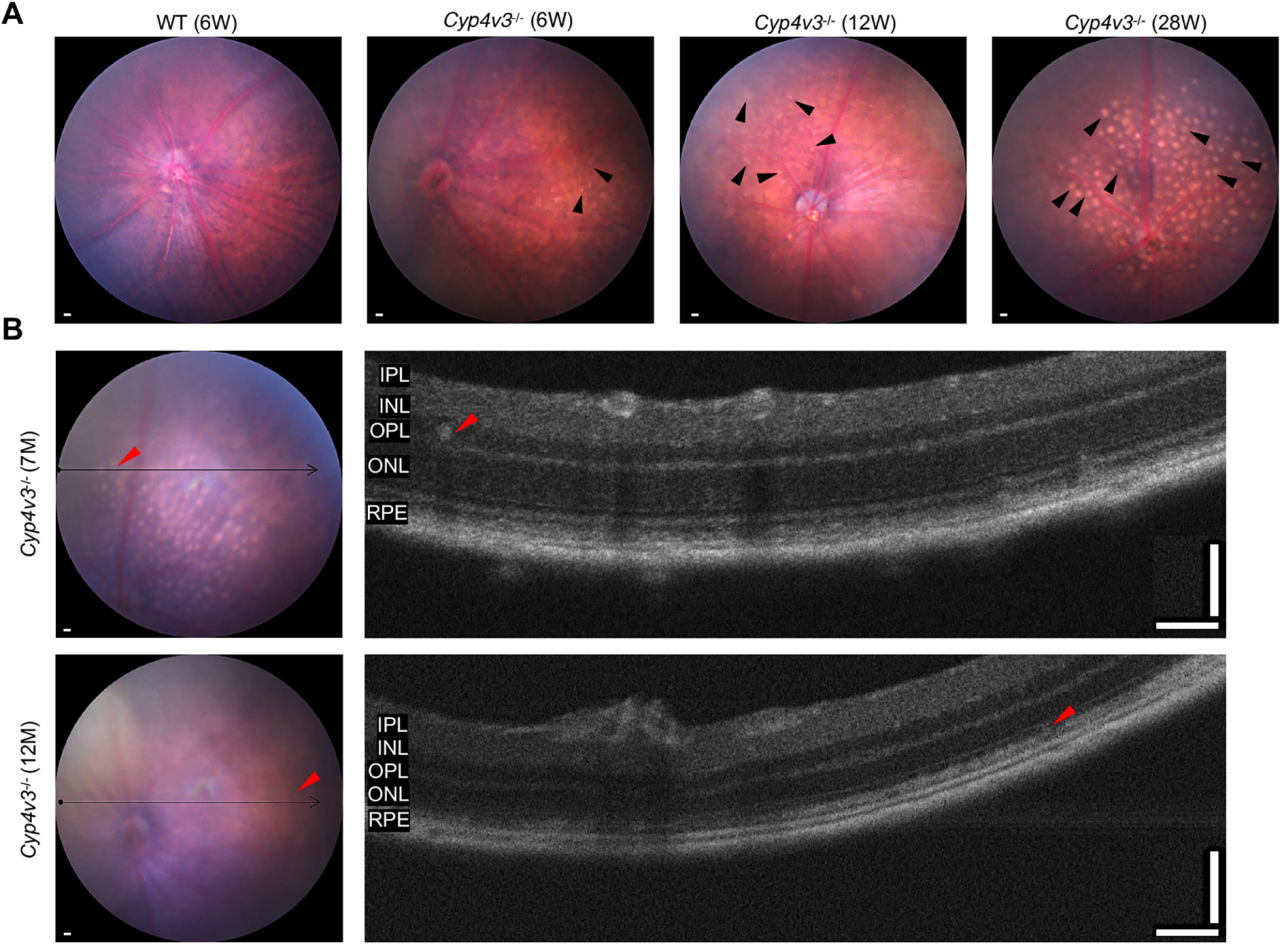
**Crystal evaluation by fundus photography and OCT in *Cyp4v3^−/−^* mice.** (A) Representative fundus images of 6-week-old wild-type (WT) mice and 6-week-old, 12-week-old and 28-week-old *Cyp4v3* knockout mice. The black arrowheads indicate crystals. Scale bars: 200 μm. (B) Representative fundus images (scale bars: 200 μm) and corresponding OCT images (scale bars: 200 μm) of *Cyp4v3* knockout mice. The thin black arrows indicate the corresponding sections between fundus and OCT. The red arrowheads indicate crystals. *n*=5 for each group.

To observe the crystalline deposits, the typical manifestation of BCD, we performed fundus imaging of *Cyp4v3^−/−^* mice (Fig. 2A). At the follow up, tiny crystal deposits were first found in *Cyp4v3^−/−^* mice at the age of 6 weeks. At 12 weeks of age, the number of crystals apparently increased. Subsequently, at 28 weeks of age, the fundus of *Cyp4v3^−/−^* mice was completely filled with large and round crystals. We performed OCT of *Cyp4v3^−/−^* mice to better understand the definite position of crystals in the retina. As shown in Fig. 2B, the crystals were present as hyperreflective foci and deposited in the superficial and deep retinal layers, which was consistent with the fundus of patients with BCD. Taken together, our novel *Cyp4v3^−/−^* mouse model exhibited an earlier process of crystalline deposits compared to the previous two mouse models under natural conditions.

### *Cyp4v3* deficiency reduced visual performance

**Fig. 3.**
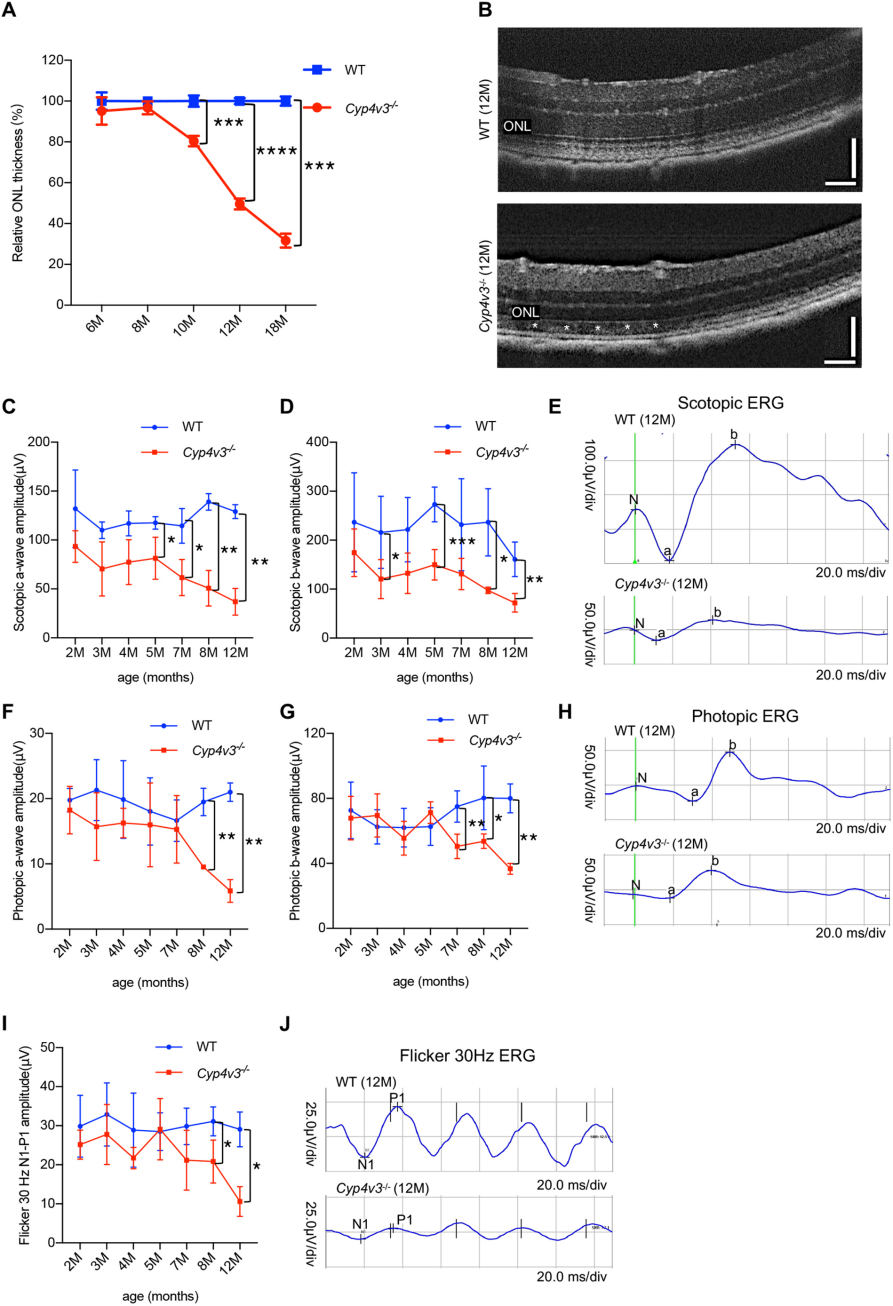
**OCT and ERG changes of *Cyp4v3* knockout mice.** (A) Quantification of the ONL thickness measured by OCT. (B) Representative OCT images of 12-month-old wild-type (WT) and *Cyp4v3^−/−^* mice. The white asterisks indicated the damage of RPE. Scale bars: 120 mm. (C) Follow up of scotopic a-wave amplitudes in wild-type and *Cyp4v3^−/−^* mice. (D) Follow up of scotopic b-wave amplitudes in wild-type and *Cyp4v3^−/−^* mice. (E) Representative wave forms of scotopic ERGs of 12-month-old wild-type and *Cyp4v3^−/−^* mice. (F) Follow up of photopic a-wave amplitudes in wild-type and *Cyp4v3^−/−^* mice. (G) Follow up of photopic b-wave amplitudes in wild-type and *Cyp4v3^−/−^* mice. (H) Representative wave forms of photopic ERGs of 12-month-old wild-type and *Cyp4v3^−/−^* mice. (I) Follow up of N1-P1-wave amplitudes of flicker 30 Hz ERG in wild-type and *Cyp4v3^−/−^* mice. (J) Representative wave forms of photopic flicker 30 Hz ERGs of 12-month-old wild-type and *Cyp4v3^−/−^* mice. Data are mean±s.d. *P<0.05, **P<0.01, ***P<0.001, ****P<0.0001 (Student’s unpaired two-tailed *t*-test). *n*=6 for each group (A-J).

As the visual function of patients with BCD is damaged after the onset, we tested the ocular functional characteristics of *Cyp4v3^−/−^* mice. Compared with wild-type mice of the same age, OCT examination showed a chronic and progressive reduction in retinal thickness, especially in the outer nuclear layer (ONL) of *Cyp4v3* knockout mice from 10 to 18 months of age (Fig. 3A). At 12 months of age, the thickness of the ONL was decreased by 50%, and the RPE was damaged (Fig. 3B). Additionally, in *Cyp4v3^−/−^* mice, the amplitude of scotopic a and b waves reduced generally from the age of 5 months, as detected by ERG functional analysis (Fig. 3C,D). The diminution in responses of photopic and flicker ERG was detected from the age of 7 months (Fig. 3F,G,I), indicating that the cones degenerated later than rods in *Cyp4v3^−/−^* mice. At 12 months of age, the responses of scotopic, photopic and flicker ERG were significantly decreased (Fig. 3E,H,J). Therefore, it suggested that the impairment of photoreceptors was accompanied by visual defect in this new mouse model.

### *Cyp4v3^−/−^* mice showed RPE and photoreceptor damage

**Fig. 4.**
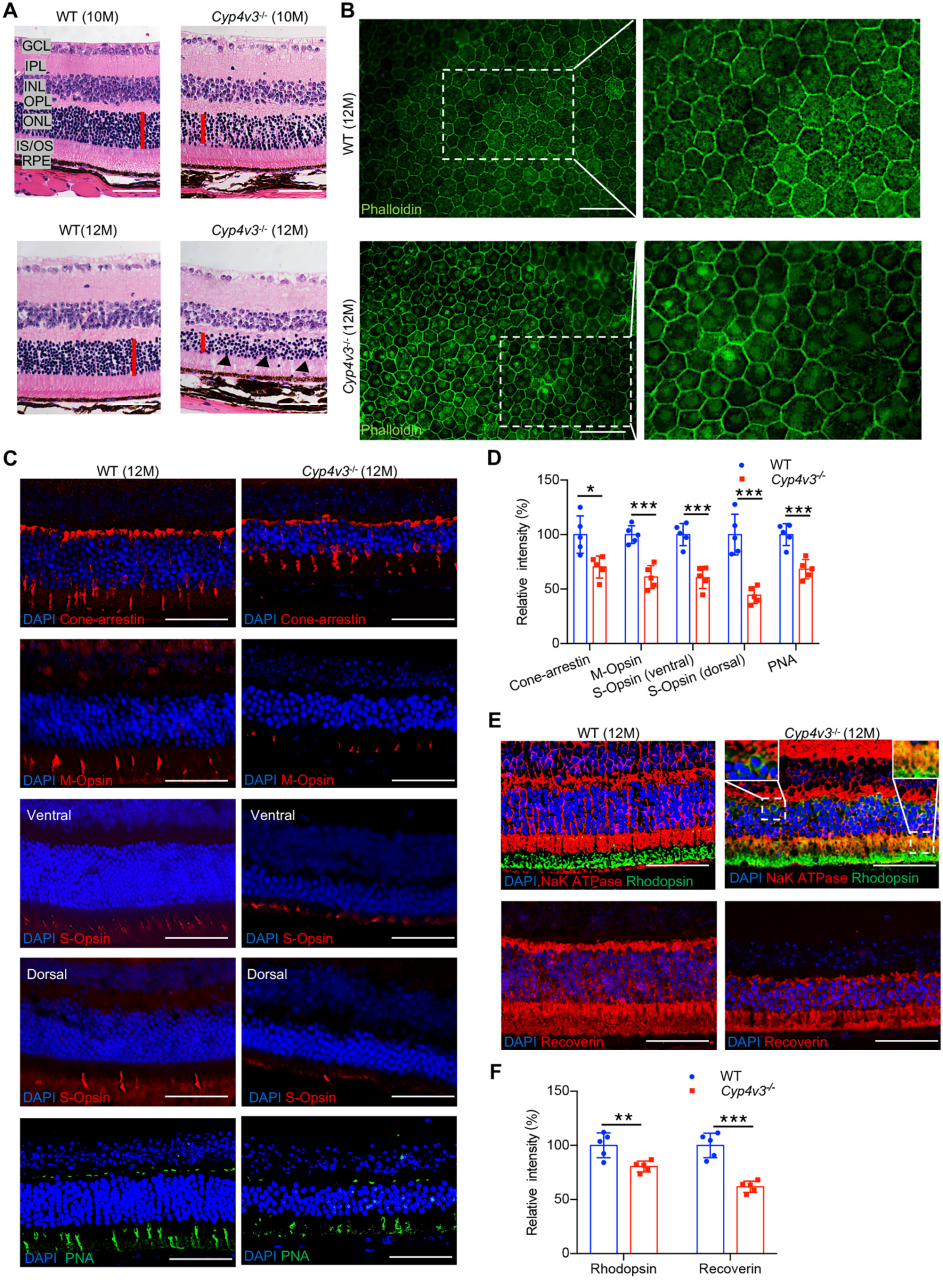
**Morphological changes of photoreceptors and RPE in *Cyp4v3^−/−^* mice.** (A) Representative H&E images of retinal sections. The black arrowheads indicate the cavity in IS/OS. The red line indicates the thickness of the ONL. (B) Phalloidin (green) staining in RPE-choroid complex flatmounts of 12-month-old wild-type (WT) and *Cyp4v3^−/−^* mice. (C) Retinal sections were stained for the remarkable markers of cone photoreceptors (Cone-arrestin, M-Opsin, S-Opsin and PNA) in 12-month-old wild-type and *Cyp4v3^−/−^* mice. (D) Densitometric analysis of the relative level of Cone-arrestin, M-Opsin, S-Opsin and PNA in 12-month-old wild-type and *Cyp4v3^−/−^* mice. (E) Retinal sections were stained for the remarkable markers of rod photoreceptors (rhodopsin and recoverin) in 12-month-old wild-type and *Cyp4v3^−/−^* mice. The inner segment was labeled by NaK ATPase (red). (F) Densitometric analysis of the relative level of rhodopsin and recoverin in 12-month-old wild-type and *Cyp4v3^−/−^* mice. Data are mean±s.d. *P<0.05, **P<0.01, ***P<0.001 (Student’s unpaired two-tailed *t*-test). Scale bars: 50 µm. *n*=5 for each group.

To obtain more evidence of the particular lesion, histological analysis was performed. Hematoxylin and Eosin (H&E) staining confirmed that the ONL of *Cyp4v3^−/−^* mice began to thin at the age of 10 months and was reduced to 50% at the age of 12 months (Fig. 4A), which was in line with the result of OCT examination (Fig. 3B). The photoreceptor cells of *Cyp4v3^−/−^* mice were atrophied, and there were many intervals between the inner segment/outer segment (IS/OS) junction (Fig. 4A). Moreover, the boundary of RPE cells was obscure in *Cyp4v3^−/−^* mice aged 12 months, as assessed by phalloidin staining (Fig. 4B), which is also consistent with the result of OCT examination (Fig. 3B).

Next, we evaluated the effect of *Cyp4v3* loss on photoreceptor structure at the age of 12 months by immunofluorescence. The density of the cone photoreceptor outer segment [labeled by cone-arrestin, M-opsin, S-opsin and peanut agglutinin (PNA)] was significantly reduced in the retina of *Cyp4v3*^−/−^ mice (Fig. 4C,D). It has been reported that M-opsin (red/green opsin) distributes evenly across the retina, whereas S-opsin (blue opsin) mainly accumulates in the ventral area (Applebury et al., 2000). As shown in Fig. S1, retinal flatmounts labeled by M-opsin or S-opsin confirmed that cone density was decreased in the *Cyp4v3*^−/−^ mice. On the other hand, the rod photoreceptor outer segment (labeled by rhodopsin or recoverin) of *Cyp4v3*^−/−^ mice was shortened and sparse compared to that of wild-type mice (Fig. 4E,F). Significantly, rhodopsin was mislocated to the inner segment (labeled by NaK ATPase) and ONL in *Cyp4v3^−/−^* retina, whereas it was located only at the outer segment in control retina (Fig. 4E). It has been well demonstrated that mislocalization and mistrafficking of rhodopsin contributed to the rod photoreceptor degeneration (Alloway et al., 2000; Nemet et al., 2015; Xiong et al., 2019). Therefore, our result might suggest that the transport of rhodopsin was dysregulated and led to the rod degeneration in the retina of *Cyp4v3^−/−^* mice. In conclusion, these findings demonstrated that the deletion of *Cyp4v3* resulted in progressive retinal degeneration.

### Analysis of lipid metabolism in the eyes of *Cyp4v3^−/−^* mice

**Fig. 5.**
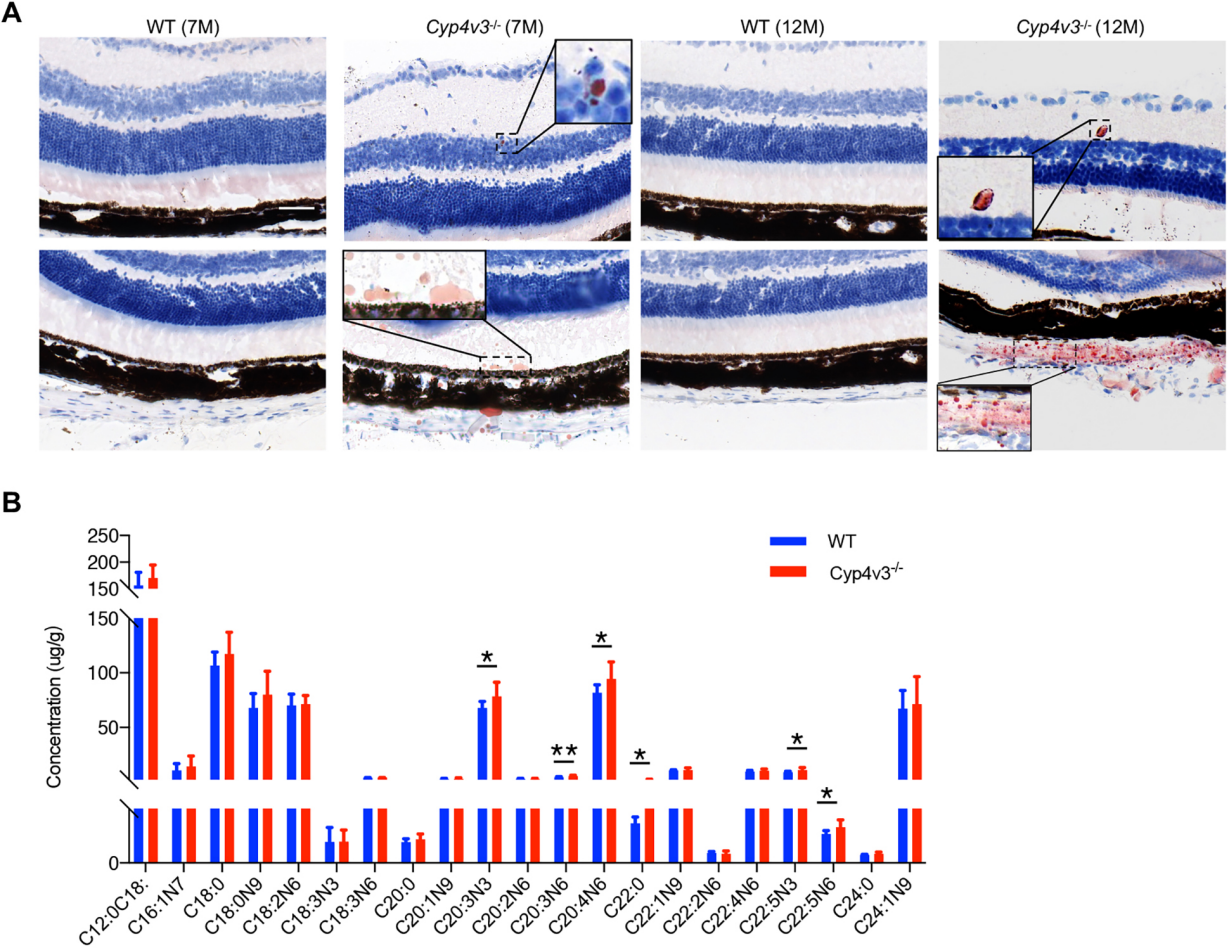
**Abnormal lipid metabolism in *Cyp4v3^−/−^* mice ocular tissue.** (A) Oil Red O staining of retinal sections in wild-type (WT) and *Cyp4v3^−/−^* mice. Scale bars: 50 µm. (B) The total FFA profile in the RPE-choroid complexes of wild-type and *Cyp4v3^−/−^* mice (aged 12 months). Data are means±s.d. *P<0.05, **P<0.01 (Student’s unpaired two-tailed *t*-test). *n*=9 for each group.

Given the role of CYP4V2 enzymes in the conversion of PUFAs, we hypothesized that the generation of crystals was related to the imbalance of lipid metabolism. Therefore, we examined the retinal section by Oil Red O staining. As shown in Fig. 5A, lipid droplets were detected in the inner nuclear layer (INL), inner plexiform layer (IPL), and IS/OS of *Cyp4v3^−/−^* mice. Furthermore, a cloud of lipid droplets was observed in the sclera of *Cyp4v3^−/−^* mice (aged 12 months), which corroborated the anomalous lipid metabolism in the ocular tissue of *Cyp4v3^−/−^* mice.

To investigate the abnormal lipid metabolism in more detail, free fatty acids (FFAs) profiling of the RPE-choroid complex of *Cyp4v3^−/−^* mice (aged 12 months) and age-matched wild-type controls was performed by GC-MS analysis. Each set comprised nine samples. Compared to wild type, the levels of C20:3N3, C20:3N6, C20:4N6, C22:0, C22:5N3 and C22:5N6 were higher in *Cyp4v3^−/−^* mice (Fig. 5B). Notably, the sera of patients with BCD showed a higher level of C20:3N6 (*P*=0.08), C20:4N6 (*P*=0.48) and C22:5N3 (*P*=0.08) than that of controls (Lai et al., 2010). This indicated some similarities between the lipid metabolism in the eyes of *Cyp4v3^−/−^* mice and the sera of patients with BCD, and these upregulated FFAs in the ocular tissue reduced by *Cyp4v3* deletion might be strongly associated with the composition of crystals.

### Transcriptome profiling by RNA-seq after *Cyp4v3* deletion

**Fig. 6.**
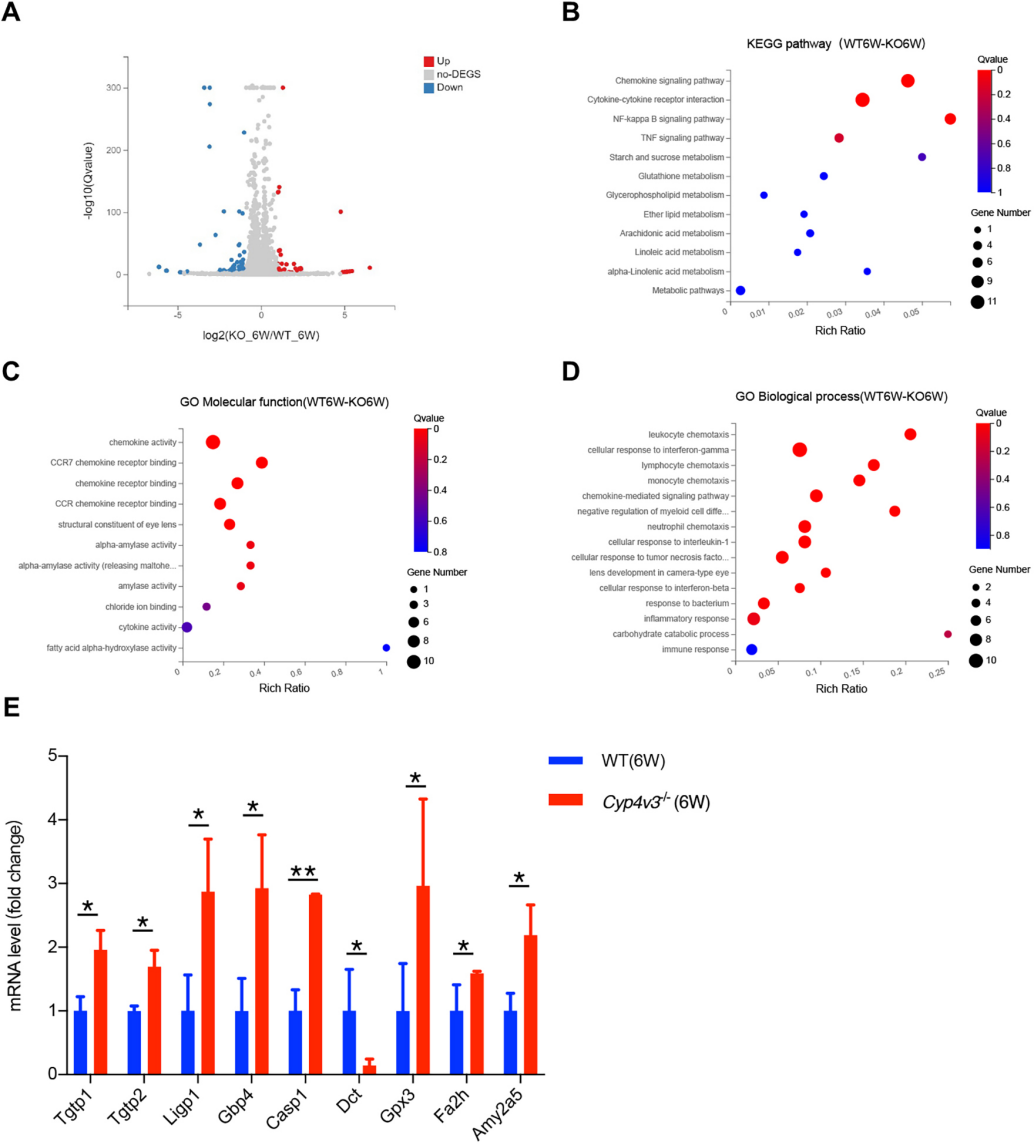
**RNA-seq analysis of 6-week-old wild-type and *Cyp4v3^−/−^* mice.** (A) Volcano plot showing meaningful changes between wild-type (WT) and *Cyp4v3^−/−^* mice. (B) KEGG pathways (adjusted *P*<0.05) in the *Cyp4v3^−/−^* mice (aged 6 weeks) and animal controls. (C) GO molecular function enrichment in *Cyp4v3^−/−^* mice (aged 6 weeks) compared with wild-type mice (adjusted *P*<0.05). (D) GO biological process enrichment in *Cyp4v3^−/−^* mice (aged 6 weeks) compared with wild-type mice (adjusted *P*<0.05). (E) Validation of gene expression by qRT-qPCR analysis. Data are means±s.d. *P<0.05, **P<0.01 (Student’s unpaired two-tailed *t*-test). *n*=6 for each group.

To find the initial molecular changes and to determine the mechanisms of progression of BCD, we performed a high-throughput RNA-seq analysis of the retinal genes in the early onset of *Cyp4v3^−/−^* mice (aged 6 weeks) and the age-matched wild-type mice. Each group contained five biological samples. By using standard criteria (fold change >2; false discovery rate <0.05), a total of 108 hits were categorized as differentially expressed genes (DEGs) between wild-type and *Cyp4v3^−/−^* mice retina, with 33 upregulated genes and 75 downregulated genes, as shown in Fig. 6A. The pathway enrichment was analyzed based on the Kyoto Encyclopedia of Genes and Genomes (KEGG) database, which showed that the DEGs were mainly enriched in pathways such as NF-kappa B signaling pathway, TNF signaling pathway, starch and sucrose metabolism, glutathione metabolism, linoleic acid metabolism, and alpha-linolenic acid metabolism (Fig. 6B). Subsequently, gene ontology (GO) enrichment analysis was also performed, which showed that chemokine activity, alpha-amylase activity and fatty acid alpha-hydroxylase activity were the most abundant groups in the molecular function category (Fig. 6C). Meanwhile, in the biological process category, cellular response to interferon-gamma, cellular response to interferon-beta and carbohydrate catabolic process were the most abundant groups (Fig. 6D). Therefore, metabolism, inflammation and oxidative stress were presumed to be involved in the initiation of crystals in *Cyp4v3* knockout mice. Moreover, metabolism associated genes [fatty acid 2-hydroxylase (Fa2h) and amylase 2a5 (Amy2a5)], inflammation associated genes [T-cell-specific GTPase 1 (Tgbp1), T-cell-specific GTPase 2 (Tgbp2) and interferon inducible GTPase 1 (Ligp1)] and oxidative stress-associated genes [guanylate binding protein 4 (Gbp4), caspase 1 (Casp1), dopachrome tautomerase (Dct) and glutathione peroxidase 3 (Gpx3)] were validated by RT-qPCR (Fig. 6E).

### The absence of *Cyp4v3* induced inflammation and oxidative stress

**Fig. 7.**
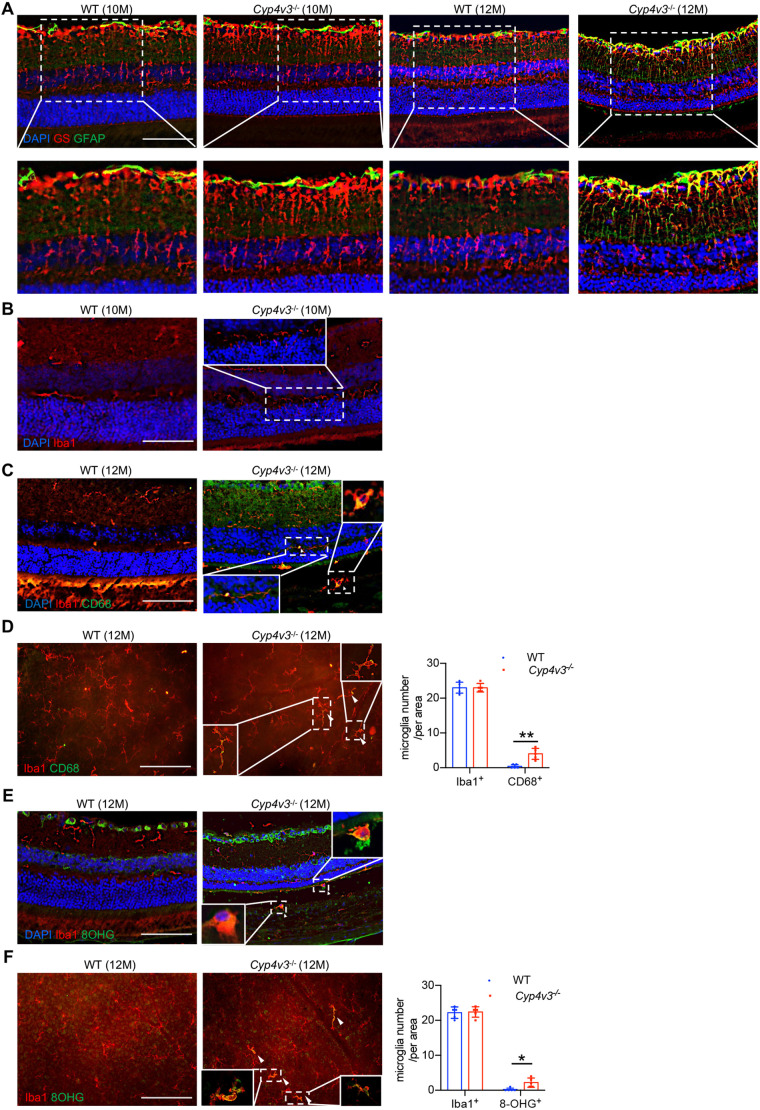
***Cyp4v3* deficiency induced retinal immune response and oxidative stress.** (A) Representative retinal sections of *Cyp4v3^−/−^* mice (aged 12 months) showing activated Müller cells (GS, red; GFAP, green) (B) Representative images of microglia cells (Iba1, red) (white arrows) with an elongated structure migrating to the ONL in the retina of *Cyp4v3^−/−^* mice (aged 10 months). (C) Representative images of activated microglia cells (Iba1, red; CD68, green) (white arrowheads) migrating to the IS/OS and RPE in the retina of *Cyp4v3^−/−^* mice (aged 12 months). (D) Retinal flatmounts of wild-type (WT) and *Cyp4v3^−/−^* mice stained for activated microglia cells (Iba1, red; CD68, green) (white arrowheads). (E) Representative images of microglia cells (Iba1, red; 8-OHG, green) (white arrowheads) experiencing oxidative stress and migrating to the IS/OS and RPE in the retina of *Cyp4v3^−/−^* mice (aged 12 months). (F) Retinal flatmounts of wild-type and *Cyp4v3^−/−^* mice stained for microglia cells experiencing oxidative stress (Iba1, red; 8-OHG, green) (white arrowheads). Data are mean±s.d. *P<0.05, **P<0.01 (Student’s unpaired two-tailed *t*-test). *n*=5 for each group. Scale bars: 100 µm.

To further verify whether inflammatory responses and oxidative stress were activated by *Cyp4v3* deletion, we performed immunofluorescence staining of *Cyp4v3^−/−^* and wild-type mice retina. In the retina of 12-month-old *Cyp4v3^−/−^* mice, Müller cells suffered from neuronal stress [labeled by glial fibrillary acidic protein (GFAP) and glutamine synthetase (GS)], with fibers extending into the ONL (Fig. 7A). Moreover, when mild atrophy occurred in the ONL, some microglia cells (labeled by Iba1) in the IPL exhibited some extended cell processes into the ONL convergently in the *Cyp4v3^−/−^* mice aged 10 months (Fig. 7B). Furthermore, the retinal sections showed that several activated microglia cells (labeled by Iba1 and CD68) were observed not only in the IPL and outer plexiform layer (OPL), but also extended into the photoreceptor outer segments and RPE in *Cyp4v3^−/−^* mice aged 12 months (Fig. 7C). From the retinal flatmounts, a significant increase was observed in the total number of CD68^+^ microglia cells (labeled by Iba1) in *Cyp4v3^−/−^* mice (Fig. 7D). Notably, immunostaining for 8-hydroxyguanine (8-OHG), a critical biomarker of oxidative stress, revealed that microglia cells experienced oxidative stress (Kawai et al., 2018). Some 8-OHG^+^ microglia cells were even located between the ONL and RPE (Fig. 7E). Additionally, compared to those of wild type, the number of 8-OHG^+^ microglia cells of *Cyp4v3^−/−^* mice evidently increased (Fig. 7F). In conclusion, the absence of *Cyp4v3* induced Müller cell- and microglia cell-mediated inflammation and oxidative stress, accompanied by a change of location of those cells in retinal layers.

### Light treatment facilitated the disease progression in *CYP4V3^−/−^* mice

**Fig. 8.**
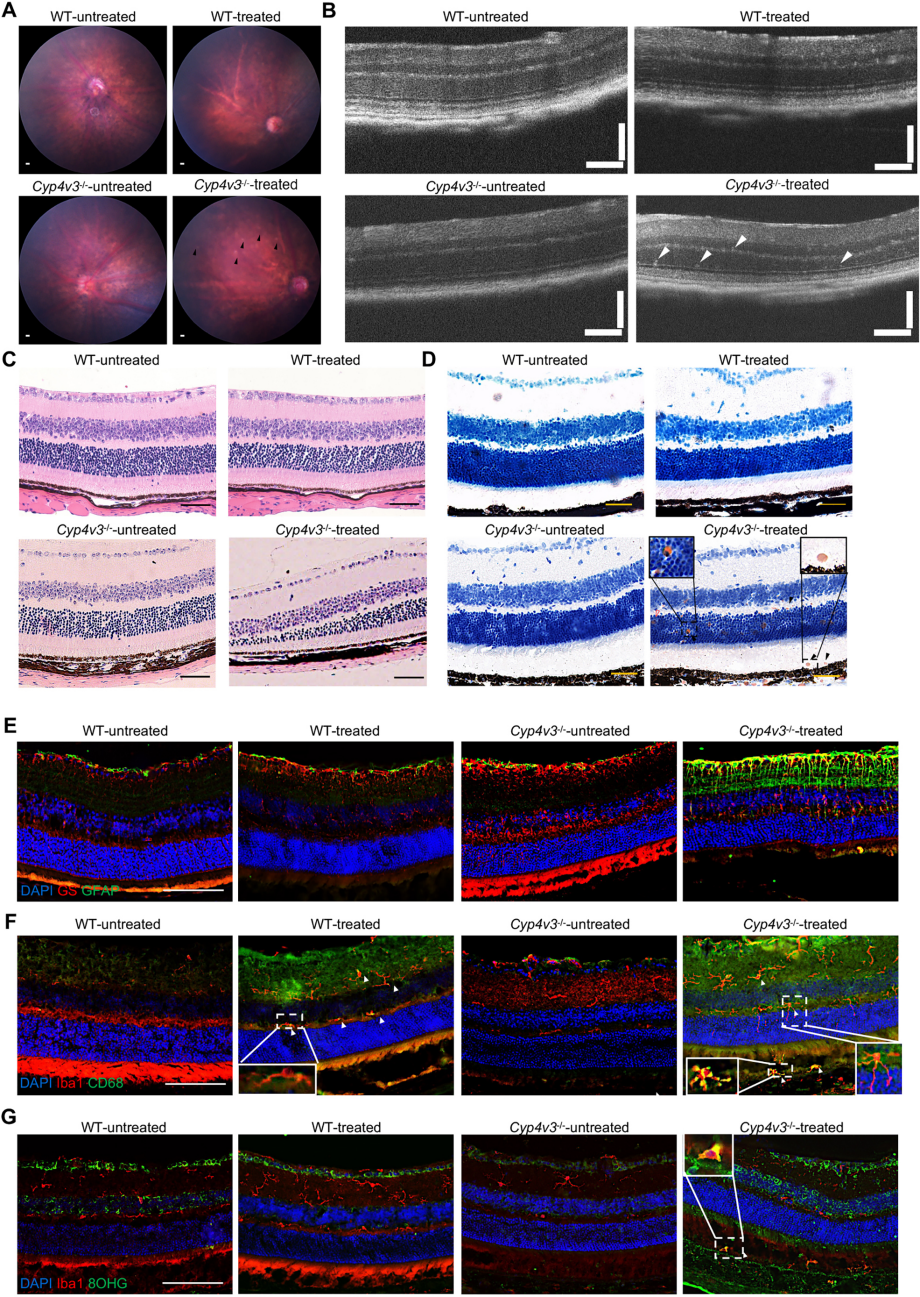
**Light treatment accelerated the disease progression of *Cyp4v3^−/−^* mouse.** (A) Representative fundus images of 6-week-old wild-type (WT) and *Cyp4v3^−/−^* mice after light treatment. The black arrowheads indicate crystals. (B) Representative OCT of 6-week-old wild-type and *Cyp4v3^−/−^* mice after light treatment. The white arrowheads indicate crystals. (C) Representative H&E images of 6-week-old wild-type and *Cyp4v3^−/−^* mice after light treatment. (D) Representative Oil Red O-stained images of 6-week-old wild-type and *Cyp4v3^−/−^* mice after light treatment. The black arrowheads indicate crystals. (E) Representative retinal sections of *Cyp4v3^−/−^* mice (aged 6 weeks) showing activated Müller cells (GS, red; GFAP, green) after light treatment. (F) Representative retinal sections of *Cyp4v3^−/−^* mice (aged 6 weeks) showing activated microglia cells (white arrowheads) (Iba1, red; CD68, green) migrating to the IS/OS and RPE after light treatment. (G) Representative images of microglia cells (Iba1, red; 8-OHG, green) (white arrowheads) infiltrating to the IS/OS and RPE following light exposure in *Cyp4v3^−/−^* mice (aged 6 weeks). *n*=6 for each group. Scale bars: 200 μm (A); 120 µm (B); 50 µm (C-G).

As immune response might contribute to the initial lesion (Natoli et al., 2016), we introduced the light treatment to monitor whether it can expedite the slow onset of BCD. Light-induced degeneration is a well-established model of retinal degeneration, including the roles of oxidative stress and neuroinflammatory activity (Natoli et al., 2016). We delivered 50,000 lux for 1 week, which could not induce any retinal damage in wild-type animals (Fig. 8A-D). In contrast, in *Cyp4v3^−/−^* mice aged 6 weeks, more crystals deposited in the fundus, accompanied with a significant decrease in retinal thickness partially, which indicated that light treatment could accelerate the progression of BCD (Fig. 8A-D). In addition, GFAP^+^ Müller cells (labeled by GS) were activated after light treatment (Fig. 8E). The microglia cells were also activated and experienced oxidative stress, and they emigrated to the outer segment and RPE (Fig. 8F,G). Therefore, the light treatment not only corroborated the role of immune response in disease progression, but it also was conducive to boosting the velocity of researching BCD.

## DISCUSSION

In the present study, we generated a novel *Cyp4v3* knockout mouse and successfully established an efficient preclinical model of BCD. This model recapitulated the characteristic features of crystalline deposits, age-related progressive photoreceptor degeneration and abnormal lipid metabolism observed in patients with BCD. Moreover, transcriptome profiling revealed that metabolism, inflammation and oxidative stress were presumed to be involved in the initiation of retinal degeneration after *Cyp4v3* deletion. Inflammation and oxidative stress mediated by microglia-Müller cells could also accelerate the progression of BCD in a LIRD mouse model of the *Cyp4v3^−/−^* mice.

Although two mouse models of BCD were reported by Lockhart et al. (2014), they could not effectively mimic the human pathological process in the ocular tissue (Lockhart et al., 2014; Qu et al., 2020). Both the models had several defects and limitations. First, the time for the emergence and prevalence of crystals in these mice was later than that in human. The former group displayed crystals that first appeared at 6 months of age and became highly confluent by 12 months of age in *Cyp4v3^−/−^* mice, whereas the latter group showed that retinal lesions of *Cyp4v3^−/−^* mice must be administered a high-fat diet. Second, the specific position of crystals in the fundus was unclear in both models. Third, retinal degeneration and changes in ERG were not reported or had no significant differences in the natural course of *Cyp4v3^−/−^* mice.

In contrast, our knockout model could recapitulate all the characteristic features of patients with BCD. The crystals were formed as early as 6 weeks of age and were fully deposited in the retina at the age of 28 weeks. The age of onset in our model was earlier and more consistent with the onset in clinical patients. Furthermore, the crystalline deposits were detected accurately as hyperreflective spots in both superficial and deep retinal layers by fundus photography and OCT, which also corresponded to the clinical manifestations in patients. Given that the crystals were widely distributed over the retina, the clinician will need to focus on the extensive visual impairment caused by the crystals. In addition, our mouse model successfully displayed age-related progressive retinal degeneration phenotypes, including a decreased ERG (from 5 months), atrophy of the ONL (from 10 months) and RPE distortion (from 12 months), which also agreed with the findings of clinical studies. Taken together, we generated a novel and more efficient mouse model of BCD.

Interestingly, different gene targeting strategies caused different phenotypes among these three knockout mice. In our experiments, the *Cyp4v3^−/−^* mice were generated using CRISPR/Cas9 technology targeting exon 1, which resulted in a 430-bp deletion and a failure in translation initiation of the entire protein. However, in the other two mice models, the entire coding region (26 kb and 28 kb) of the *Cyp4v3* gene was deleted, and exogenous gene integration (LacZ reporter and resistance cassette) was performed in one model. The Cyp4v3 protein is very simple and has no other isoforms. All these three knockout mice of *Cyp4v3* were generated in the C57BL/6J background. Excluding these factors, it remains to be answered whether deleting more sequences in the genome is better for such phenotypes.

The composition of the crystals deposited in the ocular region of *Cyp4v3^−/−^* mice has not been profiled in previous studies. First, we found that the crystals could be labeled by Oil Red O. And a subsequent GC-MS analysis showed six upregulated groups of C20:3N3, C20:3N6, C20:4N6, C22:0, C22:5N3 and C22:5N6 in the RPE-choroid complex of *Cyp4v3^−/−^* mice compared to controls. Additionally, it was reported that RPE cells play an important role in processing fatty acids and transporting them to rod inner segments (Kelly et al., 2011; Bazan, 2006). The defects of *Cyp4v3* might impair the function of RPE cells in lipid metabolism, leading to the excessive lipid accumulation in the retina. So, it may suggest that the ingredient of crystals was bound up with these upregulated FFAs.

The subsequent effects of imbalance of lipid metabolism are also worthy of investigation. On the one hand, excessive accumulation of lipids may lead to cell dysfunction or cell death, which is known as lipotoxicity (Badole and Mpharm, 2015). As we could see, the crystals deposited over the retina, which brought out the immune response. On the other hand, the generation of n-3 PUFAs was disturbed for the defects of *CYP4V2* (Nakano et al., 2012). The n-3 PUFAs included eicosapentaenoic acid (EPA) and docosahexaenoic acid (DHA), which were the key components of the photoreceptor outer segments, which were taken up by RPE cells (Fliesler and Anderson, 1983; Scott and Bazan, 1989). The PUFAs are also precursors of oxygenated resolvin and protectin metabolites that possess anti-inflammatory and immunoregulatory properties (Yin et al., 2016; Tremblay et al., 2015). As a result, the accumulation of harmful lipids and the deficiency of beneficial products will lead to the inflammation status and photoreceptor cell degeneration.

At the same time, our RNA-seq data revealed the mild inflammatory response and oxidative stress at the early stage of BCD. At the age of 12 months, *Cyp4v3^−/−^* mice showed proliferation of robust Müller cells and migration of microglial cells to the outer segment and RPE. In the retina, Müller cells act as modulators of immune and inflammatory responses, and they are the first to show metabolic changes in retinal degenerations (Tamai, 1997; Bringmann and Reichenbach, 2001). Moreover, their insufficiency has been suggested to accelerate the degeneration process (Bringmann and Reichenbach, 2001; Pfeiffer et al., 2016). Microglia, the resident immune cells in the retina, were located in the ganglion cell layer (GCL), the IPL and the OPL in healthy status (Okunuki et al., 2019). However, the accumulation of microglia in the subretinal space is considered to contribute to retinal degeneration (Alves et al., 2020). Hence, the activation of Müller cells and microglia cells uncovered the immune stress of retina and ran in parallel with the atrophy of ONL in the *Cyp4v3^−/−^* mice. Interestingly, light treatment not only triggered immune response but also promoted the lipid accumulation after *Cyp4v3* deletion. In light of these results, we can consider that impairment of lipid metabolism and the induction of immune response interacted with each other to expedite retinal degeneration. To summarize, we demonstrated the existence of retinal stress and inflammation in our model, which have not been reported in other *Cyp4v3* knockout models. Moreover, normal light treatment enhanced the process of retinal degeneration in *Cyp4v3^−/−^* mice, thus suggesting a protective benefit of avoiding light exposure for patients with BCD.

In conclusion, our study generated a novel and efficient *Cyp4v3^−/−^* mouse model, which successfully mimics the clinical symptoms of patients with BCD better than the previously published models. Additionally, our study indicated a correlation between lipid accumulation, immune response and retinal degeneration in this knockout mouse model.

## MATERIALS AND METHODS

### Construction of *Cyp4v3* knockout mice

The target ending with NGG near exon 1 was designed and cut under the action of CRISPR/Cas9. The sequence of single guide (sg)RNA1 was 5′-CCGGCAGCGACTGGTCGCCACCT-3′, and the sequence of sgRNA2 was 5′-TCCGTCTACTACTCTAACTAAGG-3′. The above sgRNA and Cas9 mRNAs were mixed by a certain concentration and proportion with a microinjection instrument, and then injected into the cytoplasm of the fertilized eggs of C57BL/6J mice *in vitro* to construct and form specific mouse embryo cells (fertilized eggs). After 1-2 h of *in vitro* culture, the surviving fertilized eggs were transplanted into the fallopian tube of the pseudopregnant female mice, and then the mice were genotyped. All mice used in our experiments listed below were male.

### Fundus photography and optical coherence tomography

Animal procedures were approved by the Shanghai Jiao Tong University Institutional Review Board, and conformed to the Association for Research in Vision and Ophthalmology Statement for the Use of Animals in Ophthalmic and Vision Research. Wild-type and *Cyp4v3* knockout animals were general anesthetized with 1% sodium pentobarbital (Millipore Sigma, Burlington, MA, USA) intraperitoneally, and were topically anesthetized with benoxinate HCl 0.4% (Santen Pharmaceuticals, Osaka, Japan) drops. We took fundus images and OCT images with a Micron IV System (Phoenix Research Laboratories, Pleasanton, CA, USA).

### Electroretinography

To assess the retinal function of *Cyp4v3* knockout mice and the littermate controls, full-field ERG was recorded using an RETIport System (Roland Consult, Brandenburg, Germany) with a Super Color Ganzfeld (Q450 SC) stimulator as described previously (Xiao et al., 2019). After dark adaptation overnight, experimental mice were anesthetized with 1% sodium pentobarbital intraperitoneally, and pupils were dilated with 1% tropicamide. The body temperature was kept at 37°C with a heating pad during the procedure. A reference electrode was placed in the center of the scalp, and a ground electrode was placed in the proximal portion of the tail skin. After the corneal surface was anesthetized using benoxinate HCl 0.4%, eye drops and contact-lens electrodes were applied directly to the corneal surface. The mouse was positioned facing the center of a Ganzfeld bowl, ensuring equal simultaneous illumination of both eyes. All the procedures were performed under dim red lighting. The scotopic ERG responses were recorded in luminance (3.0 cd×s/m^2^). After light adaption for 10 min, the photopic ERG responses were recorded in luminance (3.0 cd×s/m^2^). At the end, 30 Hz flicker ERG was recorded.

### Histology and immunofluorescence

Animals were perfused with 4% paraformaldehyde transcardially. Eyeballs were harvested for cryosections or paraffin sections. Serial paraffin sections (7 μm) were stained with H&E (ab245880, Abcam). The frozen retinal sections were stained with Oil Red O solution (ab223796, Abcam). Retina and RPE-choroid complexes were dissected out of eyeballs for flat-mounted analysis. Antibodies used for staining were anti-phalloidin (F432, Thermo Fisher Scientific, 1:500), anti-cone arrestin (ab15282, Millipore, 1:500), anti-M-opsin (red/green opsin, ab5405, Millipore, 1:500), anti-S-opsin (blue opsin, ab5407, Millipore, 1:500), anti-peanut agglutinin (FL-1071, Vector Laboratories, 1:500), anti-rhodopsin (ab98887, Abcam, 1:500), anti-NaK ATPase (14418-1-AP, Proteintech, 1:500), anti-recoverin (ab5585, Millipore, 1:500), anti-glial fibrillary acidic protein (ab4674, Abcam, 1:500), anti-glutamine synthetase (ab73593, Abcam, 1:500), anti-ionized calcium binding adapter molecule 1 (Iba1) (019-19741, Wako, 1:500), anti-8-OHG (8OHG11-M, Alpha Diagnostic International, 1:500) and anti-CD68 (MCA1957, Bio-Rad, 1:500). All primary antibodies used had been tested previously (see Supplementary Materials and Methods). The wild-type and *Cyp4v3^−/−^* mice were dark adapted more than 12 h before sacrifice, and a standard protocol (Sampath et al., 2005) was used for retinal cryosection immunofluorescence.

### RNA-seq

Retinas of 6-week-old wild-type and *Cyp4v3* knockout mice (*n*=5) were quickly separated for sequence. Total RNA was treated using the mRNA enrichment method (Liu et al., 2021). The raw data were subjected to quality control to determine whether the sequencing data were suitable for subsequent analysis. Then, sequencing was carried out using a BGIseq500 platform (BGI-Shenzhen, China).

### Sample preparation, lipid extraction and GC-MS

RPE-choroid complexes of 12-month-old wild-type and *Cyp4v3^−/−^* mice (*n*=9) were separated on an Agilent DB-WAX capillary column (30 m×0.25 mm ID×0.25 µm) gas chromatography system. A quality control sample was used for testing and evaluating the stability and repeatability of the system. MSD ChemStation software was used to extract chromatographic peak area and retention time. Medium and long chain fatty acid content in the sample was calculated by plotting the curve. The quality control samples were processed together with the biological samples.

### RNA extraction and quantitative real time PCR

Total RNA of retina was extracted using a Total RNA Extraction Kit (Tiangen, Beijing, China). RNA quantity and quality were assessed using a NanoDrop spectrophotometer (Thermo Fisher Scientific). The RNA was reverse transcribed to DNA using a cDNA Synthesis Kit (Takara Bio, Kyoto, Japan) and analyzed by quantitative real-time PCR (Applied Biosystems, Foster City, CA, USA) according to the manufacturer's instructions. Primer sequences are listed in Table S1. The ΔΔCt method was used to analyze the fold change of mRNA expression level.

### Light-induced retinal degeneration

Wild-type and *Cyp4v3^−/−^* mice of 6 weeks of age were used for the LIRD model as described previously (Liu et al., 2021). Animals were dilated with 1% tropicamide, and the mice were placed into an aluminum-foil coated cage. The cage was illuminated by a 20,000-lux white LED. Animals were provided with food and water during the time of light exposure (7 days, 8 h/day).

### Statistical analysis

Animal data are presented as mean±s.d. Student's unpaired two-tailed *t*-test was used for the comparison of results, as specified in the figure legends (Prism v7.0; Graph-Pad Software, Inc., San Diego, CA, USA). *P*<0.05 was considered statistically significant.

## Supplementary Material

Supplementary information
