# Supplementary material for: A novel and efficient murine model of Bietti crystalline dystrophy
Source: Dis Model Mech. 2022 Mar 1;15(3):dmm049222. doi: 10.1242/dmm.049222 (PMC8906172; doi:10.1242/dmm.049222)
Supplement: Supplementary information [file dmm-15-049222-s1.pdf]

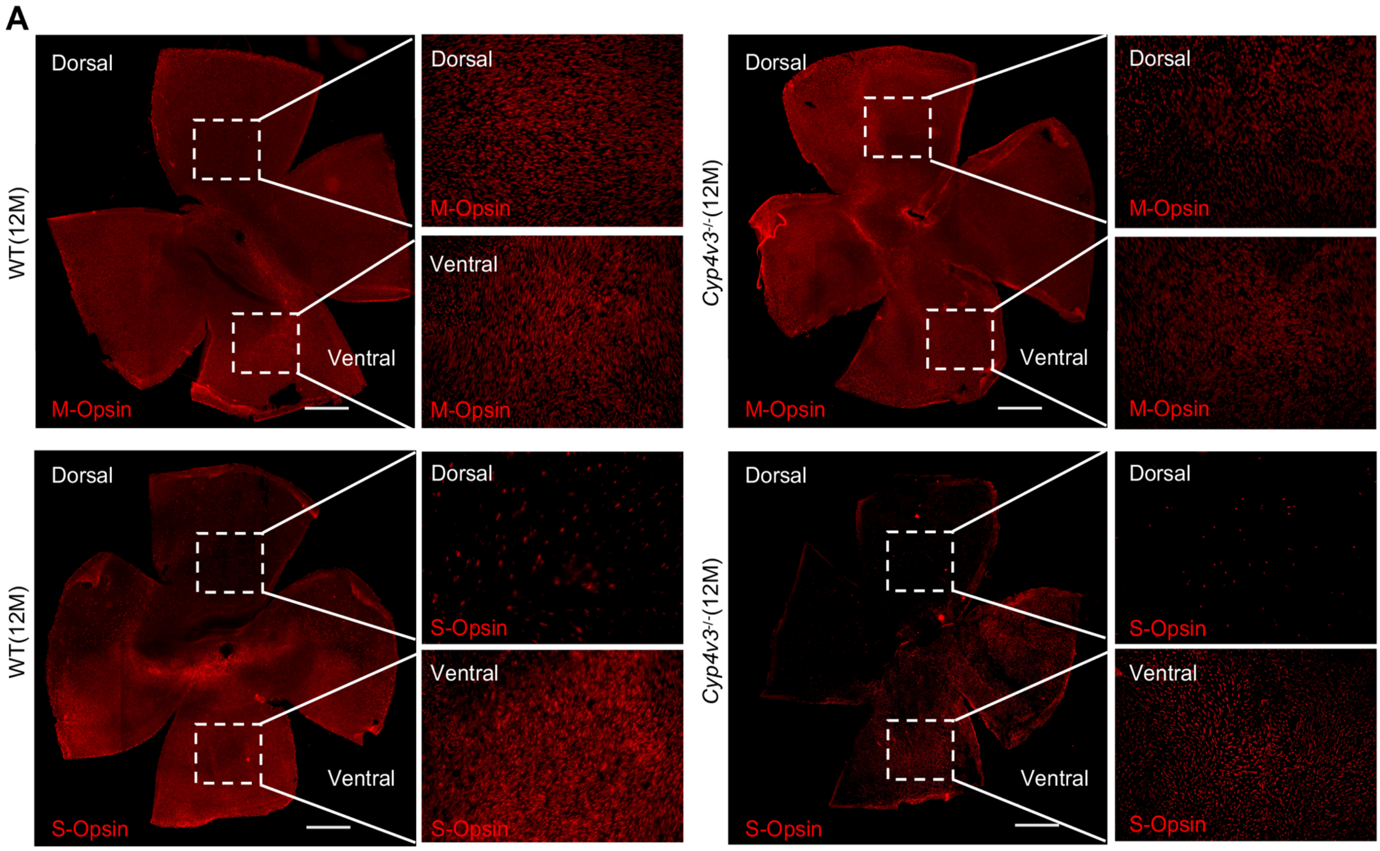


**Fig.S1. M-Opsin and S-Opsin changes of *Cyp4v3*$^{-/-}$ mice.**
(A) Representative images of retinal flatmounts immunostained with MOpsin and S-Opsin in 12-month-old WT and *Cyp4v3*$^{-/-}$ mice. Scale bars: 500 μm. n = 6 for each group.

**Table S1. The primers used for PCR**

| | **Sequence (5′-3′)** | |
|---|---|---|
| **Gene** | **Forward** | **Reverse** |
| ***GAPDH*** | ATCAAGAAGGTGGTGAAGCA | AGACAACCTGGTCCTCAGTGT |
| ***Tgtp1*** | TGCACAGATGGGGATGAATTTC | TCACTGTCGAGAGACTCCTGA |
| ***Tgtp2*** | TGGGACCACTAACTTCACACC | GGCCAGTTGTGCATCATTTTC |
| ***Ligp1*** | CAGGACATCCGCCTTAACTGT | AGGAAGTAAGTACCCATTAGCCA |
| ***Gbp4*** | GGAGAAGCTAACGAAGGAACAA | TTCCACAAGGGAATCACCATTTT |
| ***Casp1*** | ACAAGGCACGGGACCTATG | TCCCAGTCAGTCCTGGAAATG |
| ***Dct*** | TTCTGCTGGGTTGTCTGGG | CACAGATGTTGGTTGCCTCG |
| ***Gpx3*** | CCTTTTAAGCAGTATGCAGGCA | CAAGCCAAATGGCCCAAGTT |
| ***Fa2h*** | CCACTTGGGGGAGAAGTATGA | TGGGGACACTATACCAGACAG |
| ***Amy2a5*** | TTCGTACCAAGGTGGCTGAC | TGTCCACAAACACAAGGGCT |

Disease Models & Mechanisms • Supplementary information

# Supplementary Materials and Methods

## Antibody information

All primary antibodies used in our study had been tested in previous reports, including phalloidin, GS, GFAP, Iba1, CD68, 8-OHG, Rhodopsin and PNA[1],Cone Arrestin[2] , M-opsin [3] , S-opsin [4], NaK ATPase [5] and Recoverin[6].

## Reference

[1]**Liu, Y., Wang, Y., Xiao, Y., Li, X., Ruan, S., Luo, X., Wan, X., Wang, F., Sun, X.** (2021). Retinal degeneration in mice lacking the cyclic nucleotide-gated channel subunit cnga1. *The FASEB Journal*. **35**.

[2]**Kranz, K., Paquet-Durand, F., Weiler, R., Janssen-Bienhold, U. and Dedek, K.** (2013). Testing for a Gap Junction-Mediated Bystander Effect in Retinitis Pigmentosa: Secondary Cone Death Is Not Altered by Deletion of Connexin36 from Cones. *PLOS ONE* **8**, e57163

[3]**Otani, A., Dorrell, M.I., Kinder, K., Moreno, S.K., Nusinowitz, S., Banin, E., Heckenlively, J. and Friedlander, M.** (2004). Rescue of retinal degeneration by intravitreally injected adult bone marrow-derived lineage-negative hematopoietic stem cells. *J. Clin. Invest.* **114**, 765-774.

[4]**Roberts, M.R., Hendrickson, A., Mcguire, C.R. and Reh, T.A. (**2005) Retinoid X Receptor γ Is Necessary to Establish the S-opsin Gradient in Cone Photoreceptors of the Developing Mouse Retina. *Invest Ophthalmol Vis.* **46**.

[5]**Tang, Y., Fang, W., Xiao, Z., Song, M., Zhuang, D., Han, B., Wu, J. and Sun, X.** (2021). Nicotinamide ameliorates energy deficiency and improves retinal function in Cav-1$^{-/-}$ mice. *J Neurochem.* **157**, 550-560.

[6]**Gust, J. and Reh, T.A.** (2011) Adult donor rod photoreceptors integrate into the mature mouse retina. *Invest Ophthalmol Vis Sci.* **52,** 5266-72.
